# Understanding the needs and key determinants of maternal, newborn, and child health among migrants in transit: a scoping review

**DOI:** 10.1080/16549716.2025.2607905

**Published:** 2026-01-07

**Authors:** Esther Azasi, Perfect E. Asamoah, Karin Diaconu

**Affiliations:** a Institute for Global Health and Development, Queen Margaret University Institute for Global Health and Development, Musselburgh, UK; b School of Medicine, Unit of Lifespan and Population Health, University of Nottingham School of Medicine, Nottingham, UK

**Keywords:** healthcare access barriers, humanitarian health services, health systems response for migrants, social determinants of health, sexual and reproductive health

## Abstract

The global surge in migration has exposed pregnant women and children in transit to heightened risk of maternal and child health (MCH) challenges, driven by systemic barriers and unstable conditions. Evidence on how these transitory factors influence MCH remains limited. This scoping review examined the health needs and key determinants affecting migrant populations in transit, specifically pregnant women and children travelling from their countries of origin to their intended destination countries, with the aim of identifying major barriers and proposing strategies for improved health outcomes. We screened 1202 sources of evidence using five databases (PubMed, Scopus, Europe PMC, CINAHL, and Medline) as well as grey literature. Seven studies met the inclusion criteria. Data were drawn from peer-reviewed literature, charted using a standardized framework, and analysed thematically. Key barriers included financial constraints, language obstacles, and limited access to healthcare services. Although humanitarian organizations offered some support, significant unmet needs remain, including exposure to transactional sex, absence of respectful maternity care, and restricted access to essential health services. These challenges are exacerbated in conflict and crisis settings. The review underscores the importance of addressing key determinants, including location, language, financial capacity, and community support, to improve health outcomes for pregnant women and children under five on the move. This review recommends strengthening community mobilization, leveraging technology, and ensuring equitable access irrespective of users’ cultural or financial constraints.

## Paper Context

The scoping review identified key barriers to maternal, newborn, and child health among migrant populations, including financial constraints, language obstacles, and inadequate access to healthcare services.

Added knowledge The review highlights unmet needs among the study population such as the risks of transactional sex, lack of respectful maternity care, and limited access to essential services, exacerbated by the COVID-19 pandemic and the Russia–Ukraine war in some settings.

Global health impact for policy and action This review recommends enhancing community mobilization, leveraging technological innovations, and ensuring equitable access to healthcare regardless of cultural or financial barriers to improve health outcome of the study population.

## Background

Global migration has reached unprecedented levels, driven by extensive societal, political, environmental, and economic change [1,2]. In 2019, the UN estimated that 272.5 million persons migrated internationally, with half of this number being women and children [3]. Out of the 275.5 million persons, an estimated 37.9 million children were accompanied by family migrants, and around 15 million children lived out of parental care [1].

Migration disrupts the lives of individuals and compromises their ability to maintain good health, including inadequate access to consistent and quality health services. Studies have shown that women, newborns, and young children are a particularly vulnerable group [4]. For instance, a qualitative study employing ethnographic fieldwork and in-depth interviews with 25 Haitian migrants who travelled from Chile to Mexico found out that migration journeys often expose women, newborns, and children to unstable conditions, food and water insecurity, and violence, leading to severe physical and mental health challenges such as malnutrition, infections, trauma, and poor pregnancy outcome [5]. Similarly, research from South Asia [6] and other studies [7] showed that exposure to harsh environmental conditions and overcrowded, unsanitary refugee camps increase the risk of infections and maternal and child deaths among migrants.

Migrant women and children in transit (defined in this review as the temporary phase of movement between a migrant’s country of origin and their intended destination country) or on the move face significant risks due to their lack of legal status and reliance on unauthorized routes, which severely limit access to adequate healthcare in temporary shelters or travel centres [8,9]. This challenge is particularly acute in countries with already strained healthcare systems and restricted refugee rights [10]. Illegal migrant women frequently experience violence and insecurity [11]. Additional barriers such as financial constraints, language obstacles, and inadequate social support and maternity care further compromise the health and safety of this population [12–14].

Women in transit also contend with rising levels of sexual and gender-based violence, resulting in trauma, unwanted pregnancies, and sexually transmitted infections [15], while conflict and displacement limit access to safe childbirth and increase risks of maternal and neonatal deaths [13]. Children are particularly vulnerable in these situations as they are exposed to trauma and interrupted immunization schedules, which affect their cognitive development and expose them to vaccine-preventable diseases [14]. These individual challenges contribute to a broader, unresolved humanitarian crisis, as transit and host countries struggle to manage the growing influx of refugees and migrants [16].

Despite the vulnerability and complex health needs of migrant women and children, existing studies have largely focused on maternal and child health outcomes after migrants reach their destination, leaving a gap in understanding their health needs during transit [17]. Existing frameworks and guidelines, such as the Caring for Vulnerable Migrant Women [18] and Promoting the Health of Refugees and Migrants [19], primarily emphasize refugee integration and healthcare access in destination countries rather than during transit.

The lack of scientific data on the needs of migrants during transit restricts the identification of danger signs and may hinder efforts to address complications that could improve health outcomes. This lack of data is due to several factors, including the volatility of displaced communities, which makes it challenging to collect accurate data while migrants are in transit. Undocumented status further compounds this issue, especially for children [20]. Cultural and language barriers also hinder data collection, especially when health workers lack training to address the specific needs of migrants [21]. Although the WHO European Region has made recent efforts to improve the availability, accessibility, affordability, and quality of healthcare for migrants in transit and host environments [22], service provision varies across European Union (EU) member states, with some countries offering free healthcare and others requiring health insurance or contributions through employment [23,24].

Therefore, understanding the transitory health needs of migrant women and children is essential for addressing health inequalities and enhancing maternal and child health, in line with global commitments such as those outlined by the World Health Organization and Sustainable Development Goal 3 [3]. Addressing these needs is vital for achieving equitable access to healthcare and preventing increased maternal, neonatal, and postpartum health issues. Given the rising movement of migrant women, meeting their health-related needs, including reproductive health, immunisation, and mental health support, is essential for improving their well-being and fulfilling their basic human rights. This scoping review seeks to address this gap by exploring the health needs of displaced pregnant women and children during transit.

## Methodology

This scoping review followed the Arksey and O’Malley’s (2005) methodological framework, comprising five key phases: (1) identification of the research question, (2) identification of relevant studies, (3) selection of studies, (4) data charting, and (5) collation, summary, and reporting of results [25]. We also followed the Joanna Briggs Institute (JBI) recommendation of Population, Concept, and Context (PCC) for this review [26].

### Review questions

1. What are the needs of pregnant women and children during transit?
2. What are the transitory determinants of maternal, newborn, and child health outcomes during migration?
3. What factors can improve the health and well-being of pregnant women and children during transit?

Review objectives

1. To identify the health needs and transitory determinants influencing maternal, newborn, and child health (MNCH) outcomes among migrant women and children during transit.
2. To explore the factors that can enhance the health and well-being of pregnant women and children while in transit.

### Data sources and search strategy

From March to August 2024, we conducted extensive searches using both electronic databases and grey literature. To identify relevant studies, we developed detailed search strings using a combination of keywords related to ‘determinants’, ‘migration’, ‘maternal, newborn, and child health’, ‘travellers’ experience’, ‘refugees’, and ‘sexual health’. These keywords were combined using Boolean operators (e.g. AND, OR) and adapted into search strings specific to each database. We conducted forward and backward screening of all included full-text and relevant publications to identify any additional studies that fit the inclusion criteria. We also included standardized subject headings, such as Medical Subject Headings (MeSH), to ensure comprehensive coverage. The searches were conducted across five major bibliographic databases: Scopus, PubMed, CINAHL, Medline, and Europe PMC. Database searches were supplemented with manual searching of reference lists and citations until no further eligible reviews were identified. Additionally, grey literature was searched from websites such as WHO, UNICEF, the Canadian Health Research Collection, and the Canadian Research Index. No time, geographic, or language restrictions were applied.

### Inclusion and exclusion criteria

This review included migrant women and children, ensuring that the unique challenges faced by this population were thoroughly examined. In contrast, we excluded other migrant populations, such as men and smugglers, to maintain a focused analysis on women and children. Additionally, the research did not consider migrant women who were already in their destination countries, as the primary interest laid in understanding the health determinants during the migration journey itself. By adopting a worldwide perspective, the study aimed to provide a comprehensive understanding of the transitory health challenges faced by migrant women and children, highlighting the need for targeted interventions and support during their journey.

### Study selection

In total, we identified 1202 records, with 1193 sourced from databases such as PubMed, Scopus, Europe PMC, CINAHL, and Medline, and nine from additional sources via government websites, including UNICEF, the World Health Organisation, and the International Organisation for Migration. The dataset was compiled in Excel, and a deduplication command was executed, which removed 635 duplicate records, leaving 567 unique entries. Subsequently, PA conducted a title screening, eliminating 362 records due to complete irrelevance or misleading content. Titles mentioning the movement of women and children were retained and imported into Covidence software for abstract and full-text screening.

During this process, there were a few disagreements, mainly about whether the populations studied were observed while travelling (en route) or during the migration process. Most studies did not clearly specify this information, and in such cases, the studies were excluded. In addition, studies that focused on commentaries and narratives of professional advice and travellers’ perspectives regarding MNCH outcomes were excluded. These and other disagreements were resolved through detailed discussions between the reviewers. Ultimately, we unanimously excluded 116 records due to issues such as non-preferred settings, the type of article (such as professional opinions), or commentary reviews by experts, unclear populations being assessed, and misrepresented concepts. Seven studies were included in this scoping review.

### Data charting and synthesis

A predefined data extraction template was piloted and refined using the first included study before being applied to the others. The template captured key details under five main domains: Identifiers, Primary Descriptives, Methodology, Findings, and Contribution to Literature. One reviewer extracted the data, but to ensure accuracy and completeness, the other reviewer cross-checked all extracted data. Additionally, the methodological quality of all included studies was assessed using the Joanna Briggs Institute (JBI) critical appraisal tool.

**Figure 1.**
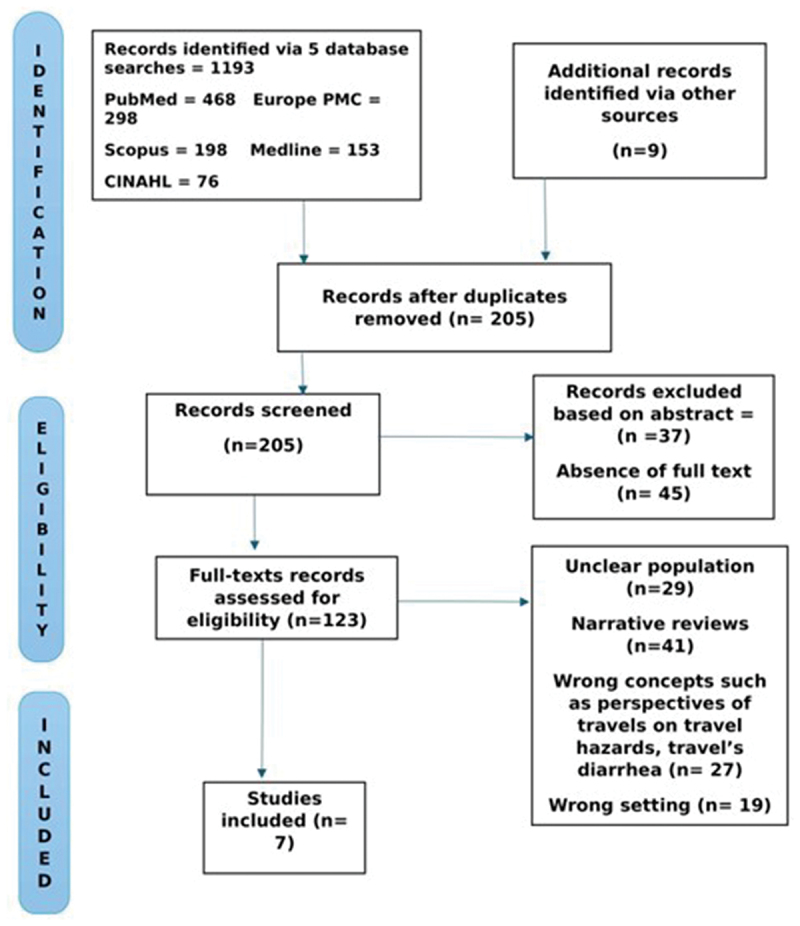
Prisma diagram showing data extraction procedure.

Source: Authors’ construct.

To summarise the findings, we followed a structured four-step approach. First, we documented the study selection process in accordance with PRISMA-SR guidelines (see Figure 1).

## Results

### Study characteristics

We identified 1202 records, of which seven were included in our analysis (see Table 1). The populations in transit examined in the scoping review were primarily pregnant women, young mothers, and adolescent girls, as highlighted in five articles [27–31]. Two additional articles focused on children; one explored the epidemiology of illness among young travellers [32], while the other provided guidance for families travelling with children [33].

**Table 1.** Description, key findings, and the needs of migrant pregnant women and children in transit.

Study	Authors	Year	Title	Country/Setting	Context	Purpose	Methodology	Key Findings	Limitations	Opportunities
Study 1	Esther Sharma, Diane Duclos, Natasha Howard	2024	The nexus between maternity care and bordering practices: A qualitative study of provider perspectives on maternal healthcare provision for Afghan women migrating through Serbia to Western Europe	Serbia	Migratory experiences of Afghan women from Serbia to Europe	Explore the perspectives and experiences of perinatal care providers to Afghan women during migration	Qualitative design using semi-structured interviews	Identified hostile public attitudes, lack of NGO support, and systemic challenges in providing maternity care	Recruitment challenges, COVID-19, geopolitical events	Non-exclusionary systems of care are needed; NGOs must adapt to changing migration governance
Study 2	Sheila Mackell	2005	Traveler’s Diarrhea in the Pediatric Population: Etiology and Impact	Developing countries	Travel sickness among children	Review the epidemiology of illness associated with travel among children	Narrative Literature Review	Children develop diarrhea at nearly the same rate as adults, treatment must address efficacy, palatability, adherence, and cost	Limited data, failed to provide literature list	Ongoing surveillance and treatments are needed, and oral rehydration solution is recommended
Study 3	Sylvia Doan, Russell W. Steele	2013	Advice for Families Traveling to Developing Countries with Young Children	USA	Young children traveling to developing countries with their parents	Provide advice for families traveling with young children using data on preventive vaccines and chemoprophylaxis	Literature review	Different treatment approaches for young children, including azithromycin and oral rehydration solutions	Limited recommendations for children, unpublished information on vaccines and medications	Guidelines for parents traveling with young children, the need for more research on vaccines and medications
Study 4	Sofya Panchenko, Philippe Mayaud, Sebastian Baranyi Nicholls, Carolina López González, Khatherine Michelle Ordáz, Madeline Baird, Amanda Gabster	2023	Sexual and reproductive health needs and sexual behaviors among migrant people in transit through Panama	Panama-Columbia Border	Sexual and reproductive health needs of migrant people in transit	Collect personal accounts of sexual behaviors and SRH needs among migrants	Rapid-assessment qualitative study using semi-structured interviews	Increased need for gynecological services, contraceptive use, and challenges for pregnant women	Limited participant diversity, reluctance to report GBV	Improved cooperation between agencies, provision of SRH services throughout the migration route
Study 5	Paola Letona, Erica Felker-Kantor, Jennifer Wheeler	2023	Sexual and reproductive health of migrant women and girls from the Northern Triangle of Central America	Northern Triangle, Central America	SRH needs of migrant women and girls	Understand SRH experiences of migrant women and girls during their journey to the USA	Descriptive, qualitative research using semi-structured interviews	High risks of transactional sex, sexual violence, and lack of access to SRH services	Lack of generalizability, challenges in accessing prenatal care	Interventions needed during predeparture phase, provision of SRH information and resources
Study 6	Michele Zaman, Victoria McCann, Sofia Friesen, Monica Noriega, Maria Marisol, Susan A. Bartel, Eva Purkey	2024	Experiences of pregnant Venezuelan migrants/refugees in Brazil, Ecuador and Peru: A qualitative analysis	Venezuela	Gendered migration experiences of Venezuelan women and girls	Understand experiences of pregnant Venezuelan migrants/refugees	Exploratory qualitative synthesis of micronarratives	Xenophobic attacks, sexual violence, lack of shelter and resources	Translation errors, convenience sampling, lack of in-depth interviews	Inform health service interventions, address xenophobia, provide access to contraception and sexual health resources
Study 7	Raquel Esther Jorge Ricart	2017	Giving birth in transit through Greece	Greece	Pregnant refugee women en route through Europe	Understand the challenges faced by pregnant refugee women during transit	Not specified	Nutrition, inadequate medical staff, inadequate sanitary conditions, poor access to camps, ignorance among younger women and those unaccompanied, paucity of translators, stigma from pregnancies resulting from rape, increase in post-traumatic stress disorders	Not specified	Highlights the severe challenges faced by pregnant refugee women, emphasizing the need for better healthcare, support, and resources

Source: Authors’ construct.

Study populations were from Afghanistan, Venezuela, and the Northern Triangle of Central America, who journeyed through Serbia, Greece, Panama, and the USA. While the primary drivers of migration were not always explicitly stated, some groups were fleeing conflict, such as Afghan women travelling through Serbia to reach Western Europe and Venezuelan women seeking refuge in Brazil, Ecuador, and Peru.

Although the studies did not provide comprehensive data on the duration of migration journeys, they showed that the journeys often involved crossing multiple countries, making prolonged stops during transit, and enduring significant hardships. A critical challenge faced by many migrant women was the lack of legal documentation, which restricted their access to healthcare and heightened their vulnerability. Some were asylum seekers or refugees with limited rights to services, while others were undocumented and entirely excluded from formal healthcare systems.

### Available support

The scoping review found that several entities, including government and non-governmental organizations, provided specific services to migrant women and children in transit. Key services provided included contraceptives and gynaecological services, intrapartum care services, antenatal and postnatal care services, nutritional and sexual and reproductive health services, emergency obstetric care, as well as mental health support services. Many of these services were provided by the UNICEF Mother and Baby corners [27]. For example, among the Afghan population in Serbia, Baby and Mother corners provided antenatal, intrapartum, and postnatal care services with special focus on infant health. When pregnant women stayed in camps, they were supported to access intrapartum care services at the facility (hospital) level. However, with some pregnant women determined to travel in their late pregnancies, some volunteers offered remote support by phoning other camp volunteers in the nearest destination to be on the lookout for pregnant clients.

The results found that gynaecological, intrapartum, and emergency obstetric care services were provided to some migrant pregnant women. For instance, UNICEF’s Mother and Baby centres in Serbia offered these essential services with the aim of supporting maternal and newborn health [27,31].

The study population also had access to some postnatal services, including breastfeeding support and immunization for newborns [27,29]. Food and nutritional recommendations were also provided to ensure children had the required nutrition for growth. For instance, one study [33] recommended supplementary feeding such as bottled or boiled water, canned fruits, and vegetables as essential for ensuring adequate nutrition. Some projects placed gynaecologists in camps to provide sexual and reproductive health services, including contraceptive and counselling sessions, to improve women’s health [28,29].

### Needs of migrants

Despite efforts to reach women and children with basic healthcare, the results showed migrant women and children in transit faced a myriad of challenges that impacted their overall health and well-being. We identified housing, security, and legal requirements as some of the general needs of women and children in transit.

#### Inadequate accommodation and poor sanitation

Accommodation and sanitary conditions were poor for most migrant populations, particularly pregnant women and their children [30,31]. Poor sanitation in asylum camps often exposed migrants to diseases, violence, and drug abuse [30]. For those exposed to sexually transmitted infections (STIs), the reviewed studies reported increased demand for gynaecological services, such as urinary tract infection (UTI) diagnosis and treatment during transit, due to unhygienic environments [31].

#### Security

Our review showed that policies concerning security, social protection, and the safeguarding of migrants in transit were inadequate. Pregnant women faced several security threats, including sexual, physical, and verbal abuse during transit [27,29,31]. Others also faced disrespectful maternity care and xenophobic attacks, including racial slurs and hostile treatment from healthcare workers while accessing care [27,31]. The findings also revealed that some were traumatised by border police or security officers during border crossings [27], and many lacked the means or courage to report their abusers [29].

### Maternal, newborn, and child health (MNCH) needs

The specific MNCH needs of migrant women and children are critical for ensuring their health and survival during transit. This review identified prenatal care and sexual and reproductive health (SRH) services as some of the needs of the study population.

#### Sexual and reproductive health

Exposure to sex during transit increased the risk of transactional sex, sexual violence, and sexually transmitted infections among women and girls, with no feasible means to report violence or abusers during the journey [29]. This underscores the need for access to contraceptives [27]. The demand for contraceptives among migrant women was linked to their need for greater control over reproductive health [28]. Nonetheless, key sexual and reproductive health (SRH) services, including injectable contraceptives and counselling, were found to be inadequate across the reviewed literature [28,29].

#### Antenatal and intrapartum care services

Pregnant migrants experienced irregular antenatal care and had inadequate knowledge of how to access maternity care [27]. Most services were perceived as lacking respectful care, interpreters and did not ask clients for their consent before certain procedures were carried out [27,29]. For example, only women who stayed in camps had some access to antenatal and maternity services [27]. Consequently, those outside camps had little to no assistance from healthcare providers during their journeys [28]. Additionally, those with access to maternity services were not involved in the service delivery processes. For instance, women had no input in certain medical procedures such as C-sections, with others lacking adequate support for breastfeeding and postnatal services [27].

None of the reviewed articles reported that healthcare providers supplied nutritional supplements to pregnant migrants. Although some women carried supplements, they were usually misplaced or stolen during the journey [28].

#### Child health – nutrition and management of childhood illnesses

The needs of migrant pregnant women and their newborns are varied yet significant for their survival and development. The findings showed that children who travelled were often susceptible to childhood diseases such as diarrhoea [32]. The article by Mackell [32], for example, argued that the best treatment choice for the paediatric traveller must address a combination of efficacy, palatability, adherence, and cost. For these reasons, infants who are not being breastfed require bottled water, canned fruits, and vegetables. Similarly, it is recommended that young children drink boiled water and freshly cooked meals [33]. The prevention and treatment of traveller’s diarrhoea in young children are approached differently. For instance, prophylaxis for high-risk circumstances or early antimicrobial therapy was not recommended for young travellers, although recommended for older children and adults. Treatment options for children now include azithromycin, along with fluid and electrolyte replacement using commercial oral rehydration solutions [33].

### Critical gaps in the delivery of MNCH services

This review showed that some of the underlying factors limiting service delivery were embedded in understaffing due to COVID-19 and inadequate resources, such as funding.

#### Inadequate staff

Mother and baby corners by UNICEF were found useful, particularly for migrant mothers, but were inadequate or weak, as most of them closed following the pandemic. The findings showed that the facilities focused mainly on infant health, with little concentration on postnatal health [27]. Moreover, contextual factors, including the outbreak of COVID-19 and the Russia–Ukraine war, further restricted the number of staff available to support women and children on the move [27]. The ‘weakening of Mother and Baby Corners, meant a lack of safe space for women’, with pregnant women feeling incapable of meeting their maternal responsibilities. It also resulted in women experiencing a sense of guilt, which negatively impacted their mental health [28].

#### Inadequate funding

Inadequate funding and investment in refugee health impacted resources available for timely and adequate services for pregnant women and their children during transit [27–32]. The review particularly found a general lack of proper shelter, resources, and financial support during the transit [31].

**Figure 2.**
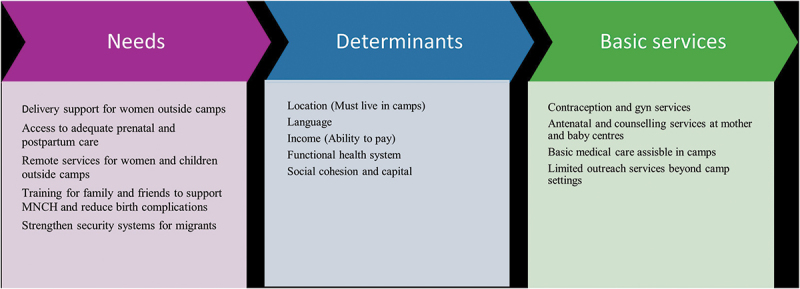
The health needs and determinants of pregnant women and children in transit.

Source: Author’s construct.

Overall, key determinants of healthcare access for women and children included location, language, and financial capacity. Figure 2 sums up the needs and determinants of migrant pregnant women and children.

The location of migrants was a key determinant of their access to RMNCH services. First, those who lived in camps had access to some resources, including intrapartum services, corner spaces, and other counselling sessions. Second, a migrant’s ability to speak the language of the host country gave them the bargaining power and some level of autonomy over some medical procedures, such as signing off on C-section procedures. Additionally, those with the ability to pay for key services either out of pocket or contribute to an existing health insurance pool increased their accessibility to healthcare. Invariably, these key services were not readily accessible to migrants outside camps.

## Discussion

Global displacement has reached unprecedented levels, driven by conflict and climate change [3,22]. Migrant women and children face significant barriers to healthcare access during transit, including financial constraints, language barriers, and inadequate maternity care [4,13,34]. These challenges pose serious risks to maternal and child health, yet little is known about their specific needs during transit. Existing literature focuses on maternal and child health outcomes after migrants reach their destination countries, leaving a gap in understanding their needs during transit. Furthermore, existing frameworks and guidelines mainly focus on migrant integration and on meeting their health needs in destination countries [21,34].

This study aimed to explore the health needs of migrant pregnant women and children in transit. Using Arksey’s framework and O’Malley Joanna Briggs Institute (JBI) recommendations, seven studies were reviewed. The results revealed that while some organizations provided contraceptives, gynaecological, and intrapartum care services such as UNICEF’s Mother and Baby Corners in Serbia, many women lacked access to essential services.

Sexual and reproductive health (SRH) rights were severely compromised. Women faced risks of transactional sex, sexual violence, and sexually transmitted infections [29]. Although some accessed contraceptives, SRH information was limited, and mechanisms to report sex offenders were absent [28,29]. Many women lacked antenatal care, respectful maternity services, interpreters, and autonomy in birthing choices [27,29]. Inadequate breastfeeding support contributed to anxiety and low self-esteem among new mothers [27,34]. Despite recommendations for supplementary feeding, none of the reviewed studies reported such support. Few of the reviewed studies explicitly examined continuity of care across antenatal, intrapartum, and postnatal stages. This gap is critical, as migration often disrupts care pathways, medical record access, and patient–provider relationships, increasing health risks for women and newborns. Strengthening cross-border coordination and introducing portable or digital health records could help maintain continuity of care for migrants on the move.

The COVID-19 pandemic and the Russia–Ukraine war further constrained service delivery by diverting staff and resources, with some informants emphasizing that empathy from host countries is vital to increasing funding and healthcare provision [27–29]. Indeed, the restriction of maternity services to women who stayed in camps raises concerns over the safety of those who gave birth during transit and outside camps [28].

The lack of adequate healthcare exposes migrant women and children to maternal and neonatal mortality, morbidity, and mental health issues. Indeed, the closure of Mother and Baby Corners affected new mothers’ ability to care for infants [28,29], whereas others faced trauma from border security personnel, with no safe reporting mechanisms [29]. These findings align with the literature on the needs of women and girls on the move [35].

The WHO’s 2017 recommendations for maternal, neonatal, and child health emphasize routine antenatal care, nutritional supplements, vaccinations, intrapartum and postnatal care, and family planning [36]. For newborns, the WHO recommends breastfeeding, immunizations, nutritional support, good hygiene, and infection control [37]. Again, the WHO’s child health guidelines stress vaccination and prevention of illnesses, including diarrhoea, pneumonia, and malaria [37]. However, this review found that services during transit fall short of these standards. Women generally lacked access to supplements, and poor sanitation increased the risks of infection and poor health.

The absence of adequate support affects the mental health and caregiving ability of most mothers. Labouring mothers outside camps risk complications like haemorrhaging and death. Newborns without immediate immunization are predisposed to preventable life-threatening conditions such as polio. Within camps, the lack of legal status and the burden of out-of-pocket payments significantly limit access to quality care. Furthermore, the decline of Mother and Baby Corners means that women no longer have a ‘safe haven’ and counselling sessions to help cope with psychological and other stressors [28]. Inadequate SRH services and protection from sexual violence increase the risks of infections like UTIs and sexually transmitted diseases (STDs), with long-term consequences.

None of the reviewed studies engaged families or communities as support systems, despite their potential capacity to provide meaningful and culturally appropriate support.

The WHO health systems framework comprises good leadership, governance, service delivery, financing, quality workforce, and information systems [35]. This review identified that challenges exist in all these areas, potentially also because health systems are focused on settled and static populations – i.e. attempting to address needs once migrant women and children reach a stable destination (e.g. camps). While in transit, migrant women and children must contend with health services and systems that suffer from underfunding, poor infrastructure, and additionally lack of empathy from host countries. Service delivery is also hindered by poor information systems and inadequate maternity care. These findings call for improved policies and resources to enhance the health of migrant women and children.

The WHO framework for the quality of maternal and newborn health care (although pertinent to the health facility level) highlights evidence-based practices for routine care and management of complications, referral systems, effective communication, emotional support, competent staff, and physical resources for improved health outcomes [38]. Contrastingly, the findings of this study reveal significant gaps across nearly all domains when measured against these parameters, underscoring the urgent need to strengthen health systems for migrant pregnant women and children.

Although none of the reviewed studies focused on African countries, the African Union (2013) in its *Multi-sector Determinants of Reproductive, maternal, Newborn and Child Health* policy brief identifies poverty, environmental conditions, socio-cultural inequalities, conflict, and insecurity as key determinants of MNCH [39]. They highlight how conflict and insecurity expose women to sexual violence, unwanted pregnancies, and disrupted livelihoods. To improve the health outcomes for this population, it is essential to implement comprehensive strategies that prioritize poverty alleviation, ensure access to nutritious food and clean water, strengthen legal protection, and promote inclusive education and political representation.

### Value of research

This research underscores the urgent need to address the basic health needs of migrant pregnant women and children irrespective of their culture, location, or financial capacity. It also identifies opportunities for stakeholders to support this population by engaging family and community structures and leveraging technology innovations to deliver targeted MNCH services during transit.

## Conclusion

This scoping review highlights the urgent need to address the health needs of migrant pregnant women and children during transit. By identifying key determinants such as *location, language, financial ability, and community support*, this study highlights critical gaps in current healthcare provisions. Improving accommodation and sanitation, increasing funding, ensuring respect for human rights, and enhancing maternal and newborn health services are essential steps to mitigate health risks for this vulnerable population.

To ensure equitable access to basic health services, mobile services, including artificial intelligence, should be considered for those outside camps. Social cohesion and capital should be leveraged to offer critical services, such as emergency delivery support during migration. Additionally, legal restrictions on providing care to women outside camps must be reviewed to allow for safe delivery.

This review calls for improved cooperation between governmental, non-governmental, and international bodies to provide a minimal package of sexual and reproductive health (SRH) prevention and care services throughout the migration route. Essential services should include gynaecological examinations, maternal and infant health services, and guidance on risk prevention for young and pregnant women in transit. Strengthening security to ensure the safety of migrant communities, particularly in host countries, is also crucial.

Further research is needed to explore the lived experiences and specific needs of migrant pregnant women during transit for improved maternity care. Addressing these gaps will help minimize preventable deaths and improve the overall health and well-being of migrant pregnant women and children.

## Limitations of the study

This study is limited by the small number of articles reviewed. As indicated in the methodology section, only seven articles were included. Additionally, none of the reviewed articles focused on African settings. Therefore, the findings of this review are not generalisable across all migrant populations, particularly those from African regions. In addition, the heterogeneity of the study designs, populations, and settings makes it difficult to draw direct comparisons or generalise results across migrant groups. The review may also be subject to publication and language bias, as studies not indexed in the selected databases or published in non-English languages might have been missed. These constraints should be taken into account when interpreting the findings and their implications for future research and policy.

## Recommendations

Women in transit require access to adequate prenatal and postpartum care. To ensure safe delivery and reduce preventable deaths, remote healthcare services should be extended to those outside formal camp settings. Additionally, strengthening cooperation between governmental, non-governmental, and international organizations is essential to deliver a core package of sexual and reproductive health (SRH) prevention along migration routes. Basic SRH services, such as menstrual hygiene products and access to clean water, should be readily available. Young and pregnant women also need targeted guidance on risk prevention, and host countries must implement policies that facilitate the reporting of gender-based violence. Essential services should include gynaecological examinations for urinary tract infections and sexually transmitted infections, as well as comprehensive maternal and infant care. Additionally, guidelines should be developed for families or individuals travelling with infants and young children, as current literature disproportionately focuses on pregnant women. Social cohesion and capital should be leveraged by engaging family members and companions travelling with pregnant women and children, particularly in managing complications during transit. This aligns with WHO recommendations to promote peer-support initiatives [33]. Finally, key stakeholders must enhance security measures to safeguard migrant communities, particularly in host countries. This includes developing anti-xenophobia policies, establishing safe spaces for survivors of gender-based violence, and safeguarding pregnant women from abuse.

## Further research

The WHO’s policy brief, ‘Improving the health care of pregnant refugee and migrant women and newborn children: policy brief’, recommends understanding the individual healthcare needs of women during the antenatal period to identify pregnancy-related risk factors and address socioeconomic barriers that may affect health outcomes [33]. As highlighted in this scoping review, very few studies have explored the maternity experiences of pregnant women during transit, limiting insight into their specific needs. Therefore, further research is recommended to investigate the lived experiences and health requirements of migrant women to inform improved maternity care during transit. Additionally, none of the articles reviewed reported that healthcare providers supplied nutritional supplements to pregnant migrants. Where supplements were carried by the women themselves, they were often lost or stolen during the journey [25].

## Supplementary Material

Table file_15Nov25.docx

Supplementary_files_2Dec25 (1)_DE.docx

Checklist file_15Nov25.docx

## Data Availability

Data sharing does not apply to this article as no data were created or analysed in this study.
