# Supplementary material for: Understanding the needs and key determinants of maternal, newborn, and child health among migrants in transit: a scoping review
Source: Glob Health Action. 2026 Jan 7;19(1):2607905. doi: 10.1080/16549716.2025.2607905 (PMC12781938; doi:10.1080/16549716.2025.2607905)
Supplement: Table file_15Nov25.docx [file ZGHA_A_2607905_SM8510.docx]

**TABLES**

**Table 1:** Description, key findings and the needs of migrant pregnant women and children in transit

| **Study** | **Authors** | **Year** | **Title** | **Country/Setting** | **Context** | **Purpose** | **Methodology** | **Key Findings** | **Limitations** | **Opportunities** |
| --- | --- | --- | --- | --- | --- | --- | --- | --- | --- | --- |
| Study 1 | Esther Sharma, Diane Duclos, Natasha Howard | 2024 | The nexus between maternity care and bordering practices: A qualitative study of provider perspectives on maternal healthcare provision for Afghan women migrating through Serbia to Western Europe | Serbia | Migratory experiences of Afghan women from Serbia to Europe | Explore the perspectives and experiences of perinatal care providers to Afghan women during migration | Qualitative design using semi-structured interviews | Identified hostile public attitudes, lack of NGO support, and systemic challenges in providing maternity care | Recruitment challenges, COVID-19, geopolitical events | Non-exclusionary systems of care are needed; NGOs must adapt to changing migration governance |
| Study 2 | Sheila Mackell | 2005 | Traveler’s Diarrhea in the Pediatric Population: Etiology and Impact | Developing countries | Travel sickness among children | Review the epidemiology of illness associated with travel among children | Narrative Literature Review | Children develop diarrhea at nearly the same rate as adults, treatment must address efficacy, palatability, adherence, and cost | Limited data, failed to provide literature list | Ongoing surveillance and treatments are needed, and oral rehydration solution is recommended |
| Study 3 | Sylvia Doan, Russell W. Steele | 2013 | Advice for Families Traveling to Developing Countries with Young Children | USA | Young children traveling to developing countries with their parents | Provide advice for families traveling with young children using data on preventive vaccines and chemoprophylaxis | Literature review | Different treatment approaches for young children, including azithromycin and oral rehydration solutions | Limited recommendations for children, unpublished information on vaccines and medications | Guidelines for parents traveling with young children, the need for more research on vaccines and medications |
| Study 4 | Sofya Panchenko, Philippe Mayaud, Sebastian Baranyi Nicholls, Carolina López González, Khatherine Michelle Ordáz, Madeline Baird, Amanda Gabster | 2023 | Sexual and reproductive health needs and sexual behaviors among migrant people in transit through Panama | Panama-Columbia Border | Sexual and reproductive health needs of migrant people in transit | Collect personal accounts of sexual behaviors and SRH needs among migrants | Rapid-assessment qualitative study using semi-structured interviews | Increased need for gynecological services, contraceptive use, and challenges for pregnant women | Limited participant diversity, reluctance to report GBV | Improved cooperation between agencies, provision of SRH services throughout the migration route |
| Study 5 | Paola Letona, Erica Felker-Kantor, Jennifer Wheeler | 2023 | Sexual and reproductive health of migrant women and girls from the Northern Triangle of Central America | Northern Triangle, Central America | SRH needs of migrant women and girls | Understand SRH experiences of migrant women and girls during their journey to the USA | Descriptive, qualitative research using semi-structured interviews | High risks of transactional sex, sexual violence, and lack of access to SRH services | Lack of generalizability, challenges in accessing prenatal care | Interventions needed during predeparture phase, provision of SRH information and resources |
| Study 6 | Michele Zaman, Victoria McCann, Sofia Friesen, Monica Noriega, Maria Marisol, Susan A. Bartel, Eva Purkey | 2024 | Experiences of pregnant Venezuelan migrants/refugees in Brazil, Ecuador and Peru: A qualitative analysis | Venezuela | Gendered migration experiences of Venezuelan women and girls | Understand experiences of pregnant Venezuelan migrants/refugees | Exploratory qualitative synthesis of micronarratives | Xenophobic attacks, sexual violence, lack of shelter and resources | Translation errors, convenience sampling, lack of in-depth interviews | Inform health service interventions, address xenophobia, provide access to contraception and sexual health resources |
| Study 7 | Raquel Esther Jorge Ricart | 2017 | Giving birth in transit through Greece | Greece | Pregnant refugee women en route through Europe | Understand the challenges faced by pregnant refugee women during transit | Not specified | Nutrition, inadequate medical staff, inadequate sanitary conditions, poor access to camps, ignorance among younger women and those unaccompanied, paucity of translators, stigma from pregnancies resulting from rape, increase in post-traumatic stress disorders | Not specified | Highlights the severe challenges faced by pregnant refugee women, emphasizing the need for better healthcare, support, and resources |

Source: Authors’ construct
