# Supplementary material for: Understanding the needs and key determinants of maternal, newborn, and child health among migrants in transit: a scoping review
Source: Glob Health Action. 2026 Jan 7;19(1):2607905. doi: 10.1080/16549716.2025.2607905 (PMC12781938; doi:10.1080/16549716.2025.2607905)
Supplement: Supplementary_files_2Dec25 (1)_DE.docx [file ZGHA_A_2607905_SM8509.docx]

**SUPPLEMENTARY FILE:**

**REVIEW SEARCH STRATEGY**

**Search terms**

1. Determinants – social factors, environmental factors, economic, weather, access to healthcare etc
2. Maternal – pregnant patients, pregnant women, mother
3. Newborn health – infant morbidity/mortality, neonatal nutrition,
4. Child health – child morbidity/mortality, childcare
5. migration – forced displacement/migration, travel, en-route, transient periods
6. health risks – health hazards, health susceptibility, health dangers, health exposure etc
7. Refugees, slaves, asylum seekers,

| Database | Search String |
| --- | --- |
| PubMed | ((((((En-route) OR ("In-transit")) AND (determinants)) OR (factors)) AND (maternal)) OR (newborn)) OR ("child health") |
|  | (((("maternal health") OR (newborn health)) OR (child health)) AND (determinants)) AND (transitory) |
|  | (((((("maternal health") OR (newborn)) OR ("child health")) AND (determinant)) OR (factors)) AND (en-route)) OR (transit) |
|  | (((((Determinant) OR (Factors)) AND ("maternal health")) OR (child)) AND (Migration)) OR (En-route) |
|  | (((((((Transitory) OR (En-route)) OR (Migration)) OR (Transit)) AND ("Maternal Health")) OR (Newborn)) OR ("Child health") |
|  | ((((((((transitory) OR (En-route)) OR (migration)) OR (transit)) AND (determinants)) OR (factors)) AND (maternal)) AND (newborn health)) AND (child health) |
|  |  |
| Scopus | “Social factors” OR “environmental factors” AND pregnant women OR mother AND infant* OR neonate* OR children AND migration OR travel OR emigration |
|  | “health risk” OR “health exposure” OR “health hazard” AND maternal OR pregnant women OR newborn OR children AND migration OR “forced displacement” |

Search terms were appropriately combined for use in Europe PMC, CINAHL, and Medline. Use this link to access a google folder for database appropriate [search histories](https://drive.google.com/drive/u/0/folders/1GP9epfMx8fbtZhLKhhYg1mXDtd1aITkA). The same terms were applied to identify relevant publications across selected grey literature sources such as WHO, UNICEF, the Canadian Health Research Collection, and the Canadian Research Index.
