# Supplementary material for: Understanding the needs and key determinants of maternal, newborn, and child health among migrants in transit: a scoping review
Source: Glob Health Action. 2026 Jan 7;19(1):2607905. doi: 10.1080/16549716.2025.2607905 (PMC12781938; doi:10.1080/16549716.2025.2607905)
Supplement: Checklist file_15Nov25.docx [file ZGHA_A_2607905_SM8508.docx]

**CHECKLIST**

**Preferred Reporting Items for Systematic reviews and Meta-Analyses extension for Scoping Reviews (PRISMA-ScR) Checklist**

| **SECTION** | **ITEM** | **PRISMA-ScR CHECKLIST ITEM** | **REPORTED ON PAGE #** |
| --- | --- | --- | --- |
| **TITLE** | | | |
| Title | 1 | Identify the report as a scoping review. | The title has identified the report as a scoping review and is documented on page 1. |
| **ABSTRACT** | | | |
| Structured summary | 2 | Provide a structured summary that includes (as applicable): background, objectives, eligibility criteria, sources of evidence, charting methods, results, and conclusions that relate to the review questions and objectives. | A structured summary (background, methods, results and conclusion) has been provided on pages 1 and 2 |
| **INTRODUCTION** | | | |
| Rationale | 3 | Describe the rationale for the review in the context of what is already known. Explain why the review questions/objectives lend themselves to a scoping review approach. | The rationale for this review is thoroughly outlined in the introduction, with a clear justification for using a scoping review approach highlighted in the final paragraph on page 5, emphasizing the need to map existing evidence on a largely underexplored issue. |
| Objectives | 4 | Provide an explicit statement of the questions and objectives being addressed with reference to their key elements (e.g., population or participants, concepts, and context) or other relevant key elements used to conceptualize the review questions and/or objectives. | Three research questions and two objectives, aligned with the Population (migrant women and children), Concept (health needs and transitory determinants), and Context (migration during transit) framework, are explicitly stated on page 6 of the manuscript. |
| **METHODS** | | | |
| Protocol and registration | 5 | Indicate whether a review protocol exists; state if and where it can be accessed (e.g., a Web address); and if available, provide registration information, including the registration number. | There is no registered protocol for this scoping review. However, a concept note is available and can be accessed if you contact the corresponding author. |
| Eligibility criteria | 6 | Specify characteristics of the sources of evidence used as eligibility criteria (e.g., years considered, language, and publication status), and provide a rationale. | On page 7, sources were eligible if they focused on the maternal, newborn, and child health (MNCH) needs of migrant women and children during transit. Studies were included regardless of publication year, language, or geographic location to ensure comprehensive global coverage. Peer-reviewed articles and grey literature were considered, while studies focusing on migrant populations already settled in destination countries, men, or smugglers were excluded. The rationale for these criteria was to maintain a focused analysis on the health determinants and needs experienced during the migration journey, a stage often underrepresented in the literature. |
| Information sources* | 7 | Describe all information sources in the search (e.g., databases with dates of coverage and contact with authors to identify additional sources), as well as the date the most recent search was executed. | A comprehensive search was conducted across five electronic databases PubMed, Scopus, Europe PMC, CINAHL, and Medline from March to August 2024 (p. 7). To enhance coverage, grey literature was also searched from reputable sources including WHO, UNICEF, the Canadian Health Research Collection, and the Canadian Research Index. Reference lists of included studies were screened for additional sources. No time, language, or geographic restrictions were applied. The most recent search was executed in August 2024. No contact was made with study authors to obtain additional information. |
| Search | 8 | Present the full electronic search strategy for at least 1 database, including any limits used, such that it could be repeated. | Full electronic search strings have been provided for PubMed and Scopus. It is attached in supplementary file No 3 on page… |
| Selection of sources of evidence† | 9 | State the process for selecting sources of evidence (i.e., screening and eligibility) included in the scoping review. | A total of 1,202 records were identified from databases and grey literature searches. After removing 635 duplicates, 567 titles were screened for relevance (p. 8). Title screening was conducted by one reviewer, and potentially relevant studies were imported into Covidence for abstract and full-text screening. Discrepancies particularly around whether studies addressed migrants in transit versus at destination were resolved through discussion between two reviewers. Studies that focused on non-target populations (e.g., men, settled migrants), opinion pieces, or poorly defined settings were excluded. Ultimately, seven studies met the inclusion criteria for this review. |
| Data charting process‡ | 10 | Describe the methods of charting data from the included sources of evidence (e.g., calibrated forms or forms that have been tested by the team before their use, and whether data charting was done independently or in duplicate) and any processes for obtaining and confirming data from investigators. | Data were charted using a predefined extraction template, which was piloted and refined using the first included study before being applied to the rest (p. 9). One reviewer conducted the initial data extraction, and a second reviewer cross-checked all extracted data to ensure accuracy and completeness. The charting process focused on five main domains: identifiers, primary descriptives, methodology, findings, and contributions to literature. No additional data was sought from study investigators. |
| Data items | 11 | List and define all variables for which data were sought and any assumptions and simplifications made. | Data was charted across five main domains, with the following variables extracted from each included study:  Identifiers – Author(s), year of publication, title, and country/region of focus.  Primary Descriptives – Study population (e.g., pregnant women, children), type of migration (e.g., in transit), and geographic context of the transit.  Methodology – Study design (qualitative, quantitative, mixed methods), sampling approach, and analytical techniques used.  Findings – Reported health needs (e.g., antenatal care, sexual and reproductive health), barriers to care (e.g., language, cost), available support services, and outcomes (e.g., SRH risks, maternal/newborn complications).  Contribution to Literature – Relevance to the review question, identification of evidence gaps, and policy implications.  Assumptions and Simplifications:  Studies were assumed to reflect health needs during transit only if explicitly stated; ambiguous timelines (e.g., "recent arrivals") led to exclusion.  Populations were included only if they involved women and children in transit; studies on men or settled migrants were excluded.  No formal imputation was made for missing data; all analyses were descriptive and thematic. |
| Critical appraisal of individual sources of evidence§ | 12 | If done, provide a rationale for conducting a critical appraisal of included sources of evidence; describe the methods used and how this information was used in any data synthesis (if appropriate). | A formal critical appraisal was not conducted, as the primary aim of this scoping review was to map the breadth and nature of available evidence rather than to assess the methodological quality or risk of bias of included studies. This is consistent with the Arksey and O’Malley framework and Joanna Briggs Institute (JBI) guidelines, which state that critical appraisal is optional in scoping reviews depending on the review’s objectives. The decision not to appraise individual sources ensured a broader inclusion of diverse evidence types relevant to understanding maternal and child health needs during migration transit. |
| Synthesis of results | 13 | Describe the methods of handling and summarizing the data that were charted. | Charted data were handled and summarized using a structured, four-step thematic synthesis approach (p. 9). After data extraction, findings from the included studies were organized into key thematic areas aligned with the review objectives. These included health needs (e.g., antenatal care, SRH services), transitory determinants (e.g., legal status, language barriers, financial constraints), and service delivery gaps. Thematic patterns were identified through comparative analysis of study contexts and populations. |
| **RESULTS** | | | |
| Selection of sources of evidence | 14 | Give numbers of sources of evidence screened, assessed for eligibility, and included in the review, with reasons for exclusions at each stage, ideally using a flow diagram. | The details of this information have been provided on the Prisma Flow Diagram which is attached as supplementary file No 2 |
| Characteristics of sources of evidence | 15 | For each source of evidence, present characteristics for which data were charted and provide the citations. | The characteristics of each source of evidence and citations have been compiled and presented in Table 1 on page 9. |
| Critical appraisal within sources of evidence | 16 | If done, present data on critical appraisal of included sources of evidence (see item 12). | Not done. Refer to Item 12 for explanation. |
| Results of individual sources of evidence | 17 | For each included source of evidence, present the relevant data that were charted that relate to the review questions and objectives. | This information can be found in Table 1 on page 9. But here is a breakdown of how each source of evidence relate to the review questions put under categories of needs, determinants and improvement factors as follows;  Sharma et al., 2024  Needs: Access to antenatal care, respectful maternity care, postnatal services.  Determinants: Camp-based support vs. exclusion outside camps, lack of interpreters, and COVID-related staff shortages.  Improvement Factors: Mother and Baby Corners, remote support coordination, camp-based referral systems.  Panchenko et al., 2023  Needs: SRH services, including contraceptives and STI treatment.  Determinants: Transactional sex exposure, poor sanitation, lack of SRH counselling.  Improvement Factors: On-site gynaecological services, SRH education.  Letona et al., 2023  Needs: Protection against sexual violence, access to respectful maternity care.  Determinants: Inadequate legal protection, xenophobia, lack of reporting mechanisms.  Improvement Factors: Family and community support, safeguarding policies.  Zaman et al., 2024  Needs: Emergency obstetric care, breastfeeding support.  Determinants: Language barriers, poor facility readiness, low service coverage.  Improvement Factors: Coordination of delivery services in camps, access to infant nutrition services.  Ricart, 2017  Needs: Safe childbirth during transit.  Determinants: Insecurity at borders, lack of maternity support on the move.  Improvement Factors: Volunteer mobilization, emergency service alerts during crossings.  Mackell, 2005  Needs: Prevention and management of childhood illnesses (e.g., diarrhoea).  Determinants: Limited access to clean water and child-friendly treatments.  Improvement Factors: Use of oral rehydration, guidance on safe feeding for young travellers.  Doan & Steele, 2013  Needs: Nutritional guidance for infants and children during travel.  Determinants: Inconsistent access to food and clean water.  Improvement Factors: Practical feeding strategies (boiled water, canned fruits/vegetables). |
| Synthesis of results | 18 | Summarize and/or present the charting results as they relate to the review questions and objectives. | Objective 1 - The review identified a range of urgent health needs, including antenatal care, respectful maternity services, sexual and reproductive health (SRH) support, mental health services, and child nutrition. Transitory determinants affecting access to care included location (camp vs. non-camp settings), language barriers, financial constraints, and legal status. Migrant women in transit often lacked legal documentation, reducing their access to healthcare and increasing their exposure to violence, unsanitary conditions, and disrespectful care (pp. 9–14). These factors severely impacted their ability to receive timely and quality maternal and newborn health services, especially outside structured camp environments.  Objective 2 - Findings highlight several enabling factors, including the role of UNICEF’s Mother and Baby Corners, which offered essential services such as antenatal care, delivery support, and immunization. Community and volunteer support networks played a role in bridging service gaps, especially when women continued travelling during late pregnancy. However, these services were inconsistently available and were severely disrupted by events like the COVID-19 pandemic and the Russia-Ukraine war (pp. 13–15). Recommendations emerging from the review include strengthening community-based support, expanding mobile health services, leveraging technology (e.g., remote support), and removing legal and systemic barriers to care for women outside formal refugee structures. |
| **DISCUSSION** | | | |
| Summary of evidence | 19 | Summarize the main results (including an overview of concepts, themes, and types of evidence available), link to the review questions and objectives, and consider the relevance to key groups. | This scoping review identified a small but diverse body of evidence (7 studies) focusing on the health needs and challenges of migrant women and children during transit. Three key themes emerged:  Health needs – including antenatal care, SRH services, respectful maternity care, mental health support, and child nutrition.  Transitory determinants – such as legal status, financial constraints, language barriers, and geographic location (inside vs. outside camps).  Support mechanisms – including camp-based services, remote volunteer coordination, and limited community-level initiatives.  These findings respond directly to the review questions and objectives by mapping both unmet needs and the barriers to care. They are relevant for humanitarian agencies, health policymakers, and service providers working along migration routes who need to understand what services are lacking and how best to reach migrant populations in transit. |
| Limitations | 20 | Discuss the limitations of the scoping review process. | This review is limited by the small number of eligible studies (n=7), which reduces the breadth of evidence.  Geographic gaps exist. No included studies were from African countries, limiting the global applicability of findings.  Many studies lacked detailed timelines, making it difficult to confirm if data strictly applied to migrants in transit.  No critical appraisal of study quality was performed, in line with scoping review methodology, but this limits conclusions about the strength of evidence. |
| Conclusions | 21 | Provide a general interpretation of the results with respect to the review questions and objectives, as well as potential implications and/or next steps. | The results confirm that pregnant women and children in transit face serious health risks, with inconsistent access to essential services like antenatal care, SRH, and child nutrition. The health of this population is shaped by transitory conditions, not just destination-level factors, a gap often missed in research and policy.  Moving forward, efforts should focus on:  Expanding mobile and remote health services along migration routes  Engaging community and family structures to support MNCH needs  Improving data collection and reporting on migrants during transit  Strengthening policies that protect migrants’ rights to health, regardless of status  These steps will help ensure vulnerable migrant populations are not left behind in global health interventions. |
| **FUNDING** | | | |
| Funding | 22 | Describe sources of funding for the included sources of evidence, as well as sources of funding for the scoping review. Describe the role of the funders of the scoping review. | This scoping review was sponsored by Queen Margaret University, Edinburgh, as part of the research sandpit project under grant number SHYNG09 IGHD |

JBI = Joanna Briggs Institute; PRISMA-ScR = Preferred Reporting Items for Systematic reviews and Meta-Analyses extension for Scoping Reviews.

* Where *sources of evidence* (see second footnote) are compiled from, such as bibliographic databases, social media platforms, and Web sites.

† A more inclusive/heterogeneous term used to account for the different types of evidence or data sources (e.g., quantitative and/or qualitative research, expert opinion, and policy documents) that may be eligible in a scoping review as opposed to only studies. This is not to be confused with *information sources* (see first footnote).

‡ The frameworks by Arksey and O’Malley (6) and Levac and colleagues (7) and the JBI guidance (4, 5) refer to the process of data extraction in a scoping review as data charting*.*

§ The process of systematically examining research evidence to assess its validity, results, and relevance before using it to inform a decision. This term is used for items 12 and 19 instead of "risk of bias" (which is more applicable to systematic reviews of interventions) to include and acknowledge the various sources of evidence that may be used in a scoping review (e.g., quantitative and/or qualitative research, expert opinion, and policy document).

*From:* Tricco AC, Lillie E, Zarin W, O'Brien KK, Colquhoun H, Levac D, et al. PRISMA Extension for Scoping Reviews (PRISMAScR): Checklist and Explanation. Ann Intern Med. 2018;169:467–473. [doi: 10.7326/M18-0850](http://annals.org/aim/fullarticle/2700389/prisma-extension-scoping-reviews-prisma-scr-checklist-explanation).
